# 4-Hydroxyisoleucine relieves inflammation through iRhom2-dependent pathway in co-cultured macrophages and adipocytes with LPS stimulation

**DOI:** 10.1186/s12906-020-03166-1

**Published:** 2020-12-09

**Authors:** Cong Zhou, Rui Chen, Feng Gao, Jiaoyue Zhang, Furong Lu

**Affiliations:** 1 Department of Integrated Traditional Chinese and Western Medicine, Union Hospital, Tongji Medical College, Huazhong University of Science and Technology, Wuhan, 430022 China; 2 Department of Endocrinology, Union Hospital, Tongji Medical College, Huazhong University of Science and Technology, Wuhan, 430022 China

**Keywords:** 4-hydroxyisoleucine, *Trigonella foenum-graecum* L., Obesity-induced inflammation, iRhom2, RAW264.7 macrophages, 3 T3-L1 adipocytes

## Abstract

**Background:**

4-Hydroxyisoleucine (4-HIL) is an active ingredient extracted from *Trigonella foenum-graecum* L., a Chinese traditional herbal medicine, which exerts the efficacy of anti-obesity and anti-diabetes. We previously reported that 4-HIL potentiates anti-inflammatory and anti-insulin resistance effects through down-regulation of TNF-α and TNF-α converting enzyme (TACE) in 3 T3-L1 adipocytes and HepG2 cells. In the present study, we further investigate the effects and mechanisms of 4-HIL on obesity-induced inflammation in RAW264.7 macrophages and 3 T3-L1 adipocytes co-culture system.

**Methods:**

RAW264.7 macrophages and 3 T3-L1 adipocytes were co-cultured to mimic the microenvironment of adipose tissue. siRNA-iRhom2 transfection was performed to knockdown iRhom2 expression in RAW264.2 macrophages. The mRNA and protein expression of iRhom2 and TACE were measured by real-time quantitative PCR (RT-qPCR) and western blotting. The production of tumor necrosis factor-α (TNF-α), monocyte chemotactic protein-1 (MCP-1), IL-6 and IL-10 were evaluated by ELISA. The ratio of M2/M1 was detected by flow cytometry.

**Results:**

4-HIL significantly repressed the mRNA and protein levels of iRhom2 and TACE in RAW264.7 macrophages after LPS stimulated. Meanwhile, the levels of pro-inflammatory cytokines, including TNF-α, MCP-1, and IL-6, were substantially suppressed by 4-HIL in the co-culture system. Moreover, the level of anti-inflammatory cytokine IL-10 was increased significantly by 4-HIL in the co-culture system after LPS stimulation. Additionally, the ratio of M2/M1 was also increased by 4-HIL in the co-culture system after LPS stimulation. Finally, these effects of 4-HIL were largely enhanced by siRNA-iRhom2 transfection.

**Conclusion:**

Taken together, our results indicated that obesity-induced inflammation was potently relieved by 4-HIL, most likely through the iRhom2-dependent pathway.

**Supplementary Information:**

The online version contains supplementary material available at 10.1186/s12906-020-03166-1.

## Background

Obesity is a medical disease that leads to many complications [1], such as diabetes, cardiovascular disease, metabolic disorders and cancer [2–5]. A body mass index (BMI) of 25.0 kg/m^2^ to 30.0 kg/m^2^ was defined as overweight; and BMI > 30.0 kg/m^2^ was defined as obesity [6]. According to WHO, worldwide obesity has nearly tripled since 1975, and there were more than 1.9 billion adults who were reported to be overweight. Among them, over 650 million were obese in 2016 [7]. Therefore, it is of great significance to explore the pathogenesis and therapeutic drugs for obesity, elucidating the pathogenesis of obesity-related metabolic diseases and finding new treatment methods. At present, there is no effective method for the treatment of obesity, and the efficacy of traditional Chinese medicine in obesity has gradually attracted widespread attention.

Obesity is characterized by a chronic state of low-degree inflammation, with macrophages gradually infiltrating into adipose tissues [8–11]. Xu H et al. identified the infiltration of macrophages in adipose tissue as the main cause of the obesity-induced inflammation [8]. It is now widely accepted that adipose tissue is the major source of many proinflammatory cytokines [12]. Proinflammatory macrophages are recruited into obese adipose tissue, causing increased attention to the interaction between immune cells and metabolism. Therefore, in this study, the Transwell system was used to co-culture RAW264.7 macrophages and differentiated 3 T3-L1 adipocyte to mimic the inflammation microenvironment of obese adipose tissue.

TNF-α is a pro-inflammatory cytokine and is considered to be a major factor in the development of obesity-induced inflammation [13]. TNF-α converting enzyme (TACE) drives macrophage homing by increasing the production of soluble TNF-α and is considered to be one of the major regulators of obesity-induced inflammation [14]. A previous study has shown a significant increase in the expression of TACE in diet-induced obese mice compared to the control group [15]. Earlier studies demonstrated that iRhom2 mediated the maturation and transport of TACE in macrophages by directly binding to TACE, and thus mediated the extracellular hydrolysis of TNF-α [16, 17]. iRhom2 gene-deficient mice exhibit reduced the levels of TNF-α [18–22]. Thus, iRhom2 might be a promising therapeutic target in the development of novel anti-diabetes and anti-obesity treatment.

4-Hydroxyisoleucine (4-HIL) is mainly extracted from *Trigonella foenum-graecum* L., accounting for about 80% of the total free amino acid content in the seeds [23]. 4-HIL is a potential insulinotropic (anti-diabetic) and anti-obesity amino acid [24]. 4-HIL has been proved to improve the secretion of glucose-dependent insulin from pancreatic cells, which is mediated by increasing Akt phosphorylation and suppressing activation of JNK, MAPK, and NF-κB, leading to reduced levels of plasma glucose, triglyceride, free fatty acid, and cholesterol [25]. Our previous study reported that 4-HIL ameliorated insulin resistance state in HepG2 cells and 3 T3-L1 adipocytes via decreasing TNF-α and TACE [26–28]. However, it still remains unknown whether 4-HIL can regulate iRhom2, which has been identified as the key factor in charge of triggering TACE-mediated TNF-α generation.

In this study, we aimed to investigate whether the co-culture of 3 T3-L1 adipocytes and RAW264.7 macrophages could aggravate inflammation, to observe the effects of 4-HIL on the co-culture system, and to explore the underlying molecular mechanisms. It has been reported that iRhom2 is mainly expressed in macrophages [16]. Hence, siRNA-iRhom2 transfection was performed to knockdown iRhom2 expression in RAW264.7 cells. Notably, we found that iRhom2 expression in RAW264.7 macrophages and 3 T3-L1 adipocytes co-culture system was inhibited by 4-HIL. Loss of iRhom2 further enhanced the anti-inflammatory effect of 4-HIL in the co-culture system. Collectively, 4-HIL relieves obesity-induced inflammation in the co-culture system of RAW264.7 macrophages and 3 T3-L1 adipocytes through iRhom2-dependent pathway.

## Methods

### Differentiation of 3 T3-L1 preadipocytes into adipocytes

The mouse 3 T3-L1 preadipocytes (CL-0006) were obtained from Procell Life Science Technology Co., Ltd. (Wuhan, China). First, 3 T3-L1 preadipocytes were seeded in 24-well plates at 5 × 10^4^ cells per well. After the cells were completely full, inducers, including 0.5 mmol/L 3-isobutyl-1-methylxanthine (I5879-100MG, Sigma, USA), 10μg/mL insulin (91077C-100MG, Sigma, USA) and 1 mmol/L dexamethasone (D1756-25MG, Sigma, USA), were added to DMEM (10,569,044, Gibco, USA) medium containing 10% FBS (#10099–141, Invitrogen, USA) to induce preadipocytes differentiation for 3 days. Then, the cells were cultured in DMEM medium containing 10 μg/mL insulin and 10% FBS for 2 days. Next, the medium was replaced with 10% FBS in DMEM medium and change once every 2 days for a total of 8–9 days. Oil red O staining indicated that 90% of the preadipocytes had acquired adipocyte morphology at day 9 (Fig. S1). All 3 T3-L1 preadipocytes need to be induced to differentiate into 3 T3-L1 adipocytes before proceeding to the next step.

### Co-culture system

The mouse macrophage cell line RAW264.7 cells (CL-0190) were obtained from Procell Life Science Technology Co, Ltd. (Wuhan, China). Mature 3 T3-L1 adipocytes and RAW264.7 macrophages were co-cultured in a Transwell system (Corning, USA) with a 0.4 μm porous membrane. After the 3 T3-L1 preadipocytes fully differentiated into mature adipocytes in a 6-well plate in the lower chamber, 5 × 10^4^ RAW264.7 macrophages were planted into the upper chamber. After 24 h, the upper chamber RAW264.7 macrophages were pretreated with 4-HIL (20 μM, ≥98.0%, 50,118-50MG, Sigma, USA) [26] 4 h before LPS (100 ng/mL, L2630-10MG, Sigma, USA) stimulation. After 6 h of LPS intervention, the total protein and nuclear protein, as well as mRNA, were then extracted from the RAW264.7 macrophages and 3 T3-L1 adipocytes. The culture supernatant was collected and stored at − 80 °C for ELISA.

### MTT assays

MTT (11,465,007,001, Sigma, USA) assay was used to detect the effects of different concentrations of 4-HIL (5 μM, 10 μM and 20 μM) on the cell viability of 3 T3-L1 adipocytes and RAW264.7 macrophages, respectively. In the non-transwell system, 3 T3-L1 preadipocytes and RAW264.7 macrophages were seeded on a 96-well cell culture plate at 5 × 10^3^ cells per well. And the cell viability of upper chamber RAW264.7 macrophages were also tested after treatment with different reagents or in different co-culture systems. The OD value of each well was detected at 490 nm using an Absorbance Reader (CMax Plus, Molecular Devices, Shanghai). Cell viability was calculated as follows: treated group OD/control group OD × 100%.

### Migration assay

The migration assay was performed in a 24-well cell culture chamber using inserts with 8 μm pores (Corning). The inserts containing 2.5 × 10^5^ RAW264.7 macrophages were transferred to wells containing 5 × 10^5^ 3 T3-L1 preadipocytes, and cultured with 4-HIL for 24 h. After culturing, the cells on the upper surface were removed. The cells on the reverse side were fixed with 70% ice-cold ethanol for 1 h, and then stained with crystal violet. Finally, the invasive cells were counted under a microscope at 200× magnification.

### Transfection of siRNA

siRNA-iRhom2 and its negative control siRNA-scrambled were synthesized (JTS, Wuhan). RAW264.7 cells were plated in 6 wells, and they were transfected with siRNA-iRhom2 or siRNA-scrambled at 30–40% cell confluence following the instruction of Lipofectamine 2000. Next, the cells were incubated in a moist incubator with 5% CO_2_ at 37 °C. After incubation for 48 h, cells were harvested. The efficiency of transfection with this siRNA pool was verified through western blotting and real-time quantitative PCR analysis of the expression of iRhom2. GAPDH was used as the loading control.

### Measurement of cytokines

Mouse TNF-α (E-EL-M0049c), MCP-1 (E-EL-M0006c), IL-6 (E-EL-M0044c) and IL-10 (E-EL-M0045c, Elabscience, Wuhan) enzyme-linked immunosorbent assay (ELISA) kits were used to measure the concentrations of TNF-α, MCP-1, IL-6, and IL-10 in the supernatant according to the manufacturer’s instructions.

### Real-time quantitative PCR analysis

After the cell culture and stimulation were completed, RAW264.7 macrophages and 3 T3-L1 adipocytes were mixed and inoculated into 9-well plates at 4 mL of medium per well. Then, mixed cells were harvested and trizol (Lot:252250AX, Aidlab, Beijing) reagent was added to obtain total RNA. Total RNA was reversely transcribed into cDNA using HiScript reverse transcriptase (R101–01/02, Vazyme, Nanjing). Under the same conditions, real-time PCR was performed on an ABI 7300 Real-Time PCR instrument using the SYBR Green Master Mix (Q111–02, Vazyme, Nanjing) system: 50 °C for 2 min, 95 °C for 10 min, 95 °C for 30 s, and 60 °C for 30 s, 40 cycles. Finally, a dissolution curve was drawn and the 2^^(−△△Ct)^ method was used to analyze the final data. GAPDH was used as the internal reference. The following gene primer sequences were obtained from NCBI (Bethesda, USA):

**Table:** 

GAPDH	(forward) 5′-ATGGGTGTGAACCACGAGA-3′;
	(reverse) 5′-CAGGGATGATGTTCTGGGCA-3′.
iRhom2	(forward) 5′-AGAACAGAGGCGTGTATGAGAG-3′;
	(reverse) 5′-CCAGTATCATTCTGCCACTTTACGA-3′.
TACE	(forward) 5′-TGAGGAAAGGGAAGCCATGT −3′;
	(reverse) 5′- ACCAGAACAGACCCAACGAT −3′.

### Western blot analysis

Cell culture and stimulation were described above. RAW264.7 macrophages and 3 T3-L1 adipocytes were mixed and washed with cold PBS and lysed with RIPA lysate (P0013B, Beyotime, Shanghai) to extract total protein. BCA protein concentration assay kit (P0010, Beyotime, Shanghai) was used to measure protein concentration. Next, the protein samples were loaded onto 5% or 12% sodium dodecyl sulfate-polyacrylamide gel electrophoresis (SDS-PAGE) gels (NP0002, NuPAGE, USA) and then transferred to a 0.45 μm PVDF membrane (88,585, Thermo Fisher, USA). The PVDF membrane was immersed in TBST (blocking solution) containing 5% skimmed milk powder and blocked at room temperature on a shaker for 2 h, then incubated with specific primary antibodies overnight at 4 °C. The PVDF membrane was immersed in the secondary antibody incubation solution. After washing, ECL reagent and the stable peroxidase solution were mixed at 1:1 ratio, then added to the PVDF membrane, and the gray value of the film was analyzed with Band Scan after X-ray film (Carestream, Xiamen, China) compression and standardized to GAPDH and expressed as a loading control.

### Flow cytometry

The upper chamber RAW264.7 macrophages were washed twice with cold PBS buffer. After centrifugation, the supernatant was removed and the pellet was resuspended in PBS buffer. Then, cells were incubated with F4/80 monoclonal antibody (17–4801-80, Invitrogen, USA) (APC conjugated), CD11c monoclonal antibody (FITC conjugated) (11–0114-81, Invitrogen, USA), and CD11b monoclonal antibody (12–0112-81, Invitrogen, USA) (PE-conjugated). Cells were analyzed on a CytoFLEX Cell Analyzer (Beckman, USA) with post-processing in CytExpert software.

### Statistical analysis

All data were presented as mean ± standard deviation (SD), analyzed by SPSS version 23.0 software and graphed by GraphPad Prism version 7 software. Differences between groups were analyzed by one-way ANOVA and Student’s t-test. *P* values less than 0.05 were considered to indicate significance.

## Results

### 4-HIL has no obvious toxicity on RAW264.7 cells and adipocytes

**Fig. 1:**
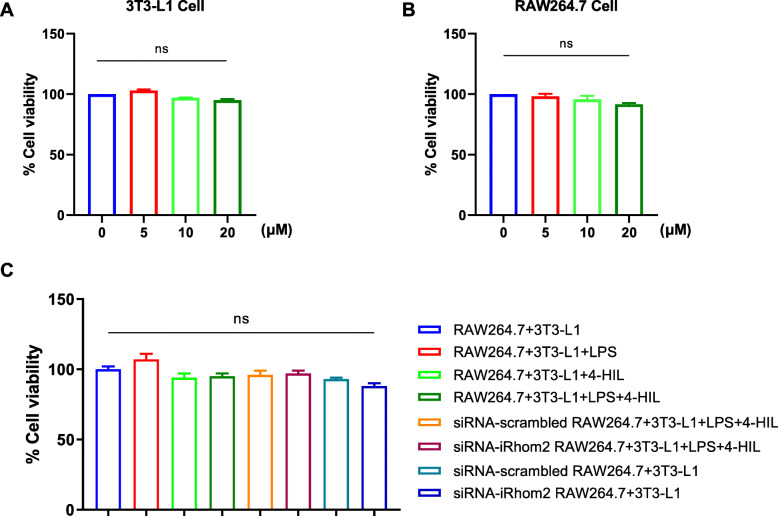
4-HIL is non-toxic to the co-culture system. The RAW264.7 cells and 3 T3-L1 cells or co-culture system were treated with 4-HIL (20 μM) and LPS (100 ng/ml) for 6 h. **a** The cell viability of 3 T3-L1 cells. **b** The cell viability of RAW264.7 cells. **c** The cell viability of RAW264.7 in co-culture system

MTT was used to detect the effects of 4-HIL and other reagents on the cell viability of the co-culture system. As shown in Fig. 1a-b, different concentrations of 4-HIL have no significant effect on the viability of 3 T3-L1 cells and RAW264.7 cells after 4 h, respectively. And, as shown in Fig. 1c, no significant difference in cell viability was observed after 4 h of different interventions (*P* > 0.05, 4-HIL, LPS, LPS + 4-HIL, siRNA-scrambled + LPS + 4-HIL, siRNA-iRhom2 + LPS + 4-HIL, siRNA-scrambled, siRNA-iRhom2).

### The expression of iRhom2 is inhibited after siRNA-iRhom2 transfection in RAW264.7 cells

**Fig. 2:**
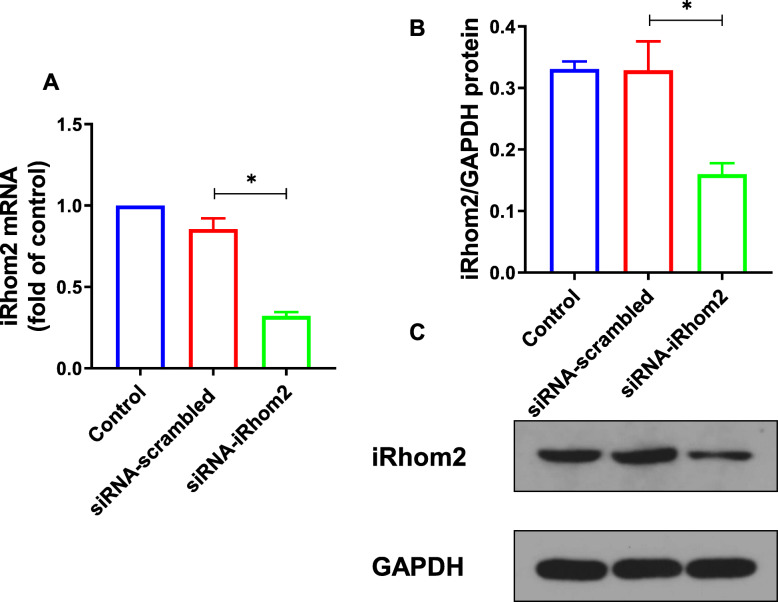
The expression of iRhom2 is inhibited after siRNA-iRhom2 transfection in RAW264.7 cells. RAW264.7 cells were transfected with siRNA-iRhom2 or siRNA-scrambled. Twenty-four hours after transfection, the expression of iRhom2 was measured. **a** The mRNA level of iRhom2 detected by RT-qPCR. **b** Densitometric analysis of iRhom2. **c** The expression of iRhom2 measured by Western blot. Quantitative data were presented as mean ± SD. ^*^P < 0.05

iRhom2 is mostly expressed in immune cells, especially macrophages, while its expression in other cells is very low, or even not expressed [16]. In order to knock down the expression of iRhom2, siRNA-iRhom2 was transfected into RAW264.7 macrophages. And siRNA-scrambled was used as a negative control. Then, the mRNA and protein expression of iRhom2 in RAW264.7 macrophages were analyzed by RT-qPCR and western blotting (Fig. 2). Twenty-four hours after transfection, iRhom2 expression was significantly inhibited by siRNA-iRhom2 in RAW264.7 macrophages (*P* < 0.05).

### 4-HIL downregulates the expression of iRhom2 and TACE in the co-culture system with LPS induction

**Fig. 3:**
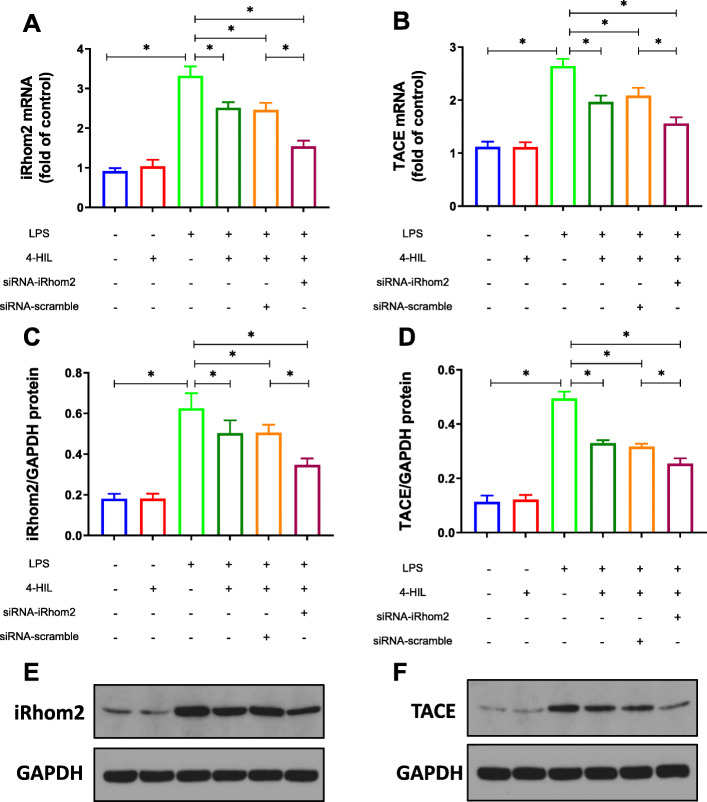
4-HIL downregulates the expression of iRhom2 and TACE in the co-culture system induced by LPS. **a** The mRNA level of iRhom2 detected by RT-qPCR. **b** The mRNA level of TACE detected by RT-qPCR. **c** Densitometric analysis of iRhom2. **d** Densitometric analysis of TACE. **e** The expression of iRhom2 measured by Western blot. **f** The expression of TACE measured by Western blot. Quantitative data were presented as mean ± SD. ^*^P < 0.05

Our previous studies confirmed that the anti-inflammatory property of 4-HIL was dependent on the down-regulation of TACE and TNF-α in 3 T3-L1 adipocytes and HepG2 cells [26–28]. In this study, the mRNA and protein expression of iRhom2 and TACE in the co-culture system was analyzed by RT-qPCR and western blotting (Fig. 3). Our results showed that the mRNA and protein expression of iRhom2 and TACE were significantly increased in the co-culture system (*P* < 0.05) after 6 h LPS stimulation. Meanwhile, this LPS-induced iRhom2 and TACE expression was noticeably blocked by 4-HIL (*P* < 0.05). Subsequently, these effects of LPS were significantly abrogated by siRNA-iRhom2 transfection in RAW264.7 cells (*P* < 0.05). Interestingly, 4-HIL had little or no effect on iRhom2 and TACE without LPS stimulation (*P* > 0.05).

### 4-HIL attenuates the levels of TNF-α and MCP-1 in the co-culture system with LPS stimulation in a dose- and time-dependent manner

**Fig. 4:**
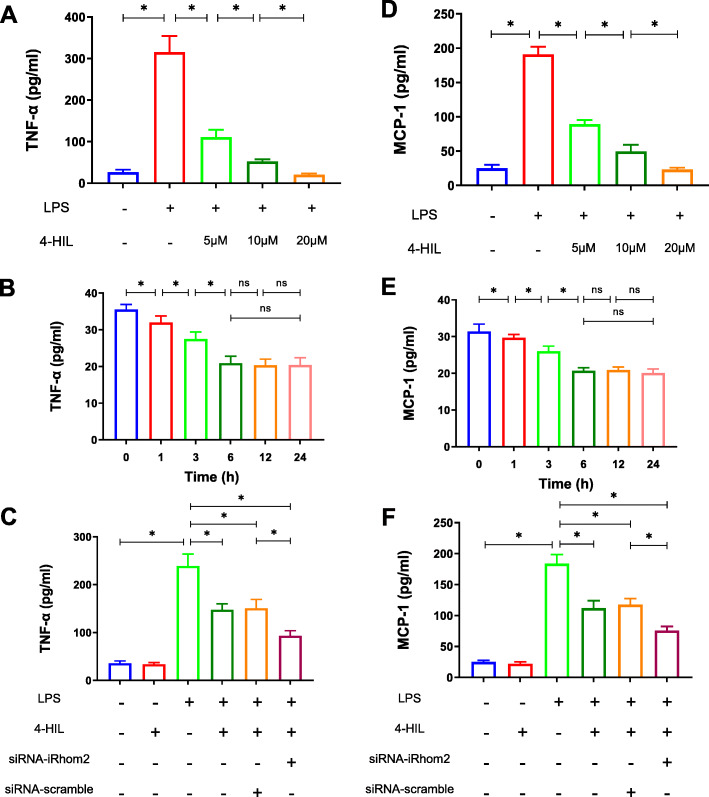
4-HIL attenuates the levels of TNF-α and MCP-1 in a time- and dose-dependent manner in the LPS-induced co-culture system. **a-c** The levels of TNF-α tested by ELISA. **d**-**f** The levels of MCP-1 tested by ELISA. Quantitative data were presented as mean ± SD. *P < 0.05

There is evidence showing that TNF-α and MCP-1 are involved in inflammation, which was induced by the interaction of adipocytes and macrophages in obese adipose tissue [29]. In this study, ELISA was used to determine the effects of 4-HIL on the levels of TNF-α and MCP-1 (Fig. 4). After 6 h of LPS stimulation, the levels of TNF-α and MCP-1 were reduced by 4-HIL in a dose-dependent (Fig. 4a and d) and time-dependent manner (Fig. 4b and e). Meanwhile, those effects of LPS was markedly abrogated by siRNA-iRhom2 transfection in RAW264.7 cells (Fig. 4c and f, *P* < 0.05). However, 4-HIL had little or no effect on the production of TNF-α and MCP-1 without LPS stimulation (*P* > 0.05).

### 4-HIL suppresses macrophage migration, promotes M2 macrophage polarization, and inhibits M1 macrophage polarization in the co-culture system with LPS stimulation

The infiltration of macrophages in adipose tissue was identified as the main cause of the obesity-induced inflammation [8]. M1 macrophages release pro-inflammatory cytokines, such as IL-6, which can directly promote the migration of macrophages and further promote obesity-induced inflammation. In contrast, M2 macrophages secrete anti-inflammatory cytokines, such as IL-10, which suppress inflammation and have a positive effect on improving insulin resistance [30–32].

**Fig. 5:**
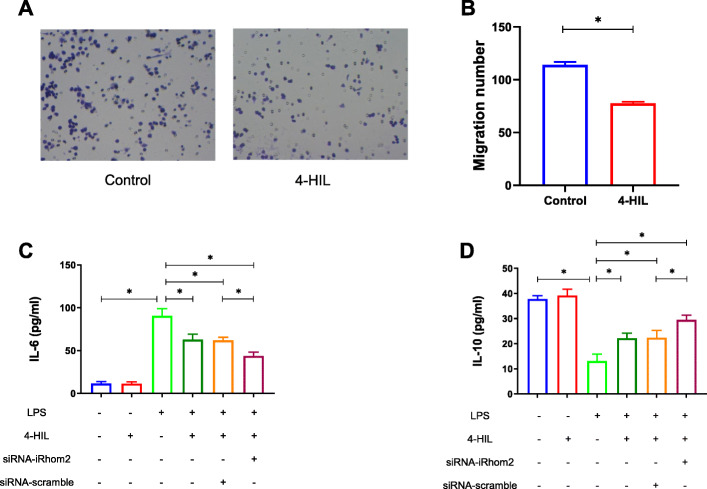
4-HIL reduces the levels of pro-inflammatory cytokines IL-6 and increases the levels of anti-inflammatory cytokines IL-10. **a** RAW264.7 macrophage crystal violet stain (200X). **b** Quantification of RAW264.7 macrophage migration number. **c** The levels of IL-6 tested by ELISA. **d** The levels of IL-10 tested by ELISA. Quantitative data were presented as mean ± SD. ^*^P < 0.05

As shown in Fig. 5a-b, 4-HIL significantly inhibited the migration of RAW264.7 macrophages in 3 T3-L1 adipocytes. Next, we determined the levels of IL-6 and IL-10 (Fig. 5c-d). Our findings suggested that the product of M1 macrophage IL-6 were significantly increased and the product of M2 macrophage IL-10 was significantly decreased in the co-culture system (*P* < 0.05) after 6 h of LPS stimulation. Meanwhile, this LPS-induced production of IL-6 and IL-10 were noticeably blocked by 4-HIL (*P* < 0.05). Subsequently, these effects of LPS were significantly abrogated by siRNA-iRhom2 transfection in RAW264.7 macrophages (*P* < 0.05). Interestingly, 4-HIL had little or no effect on IL-6 and IL-10 without LPS stimulation (*P* > 0.05).

**Fig. 6:**
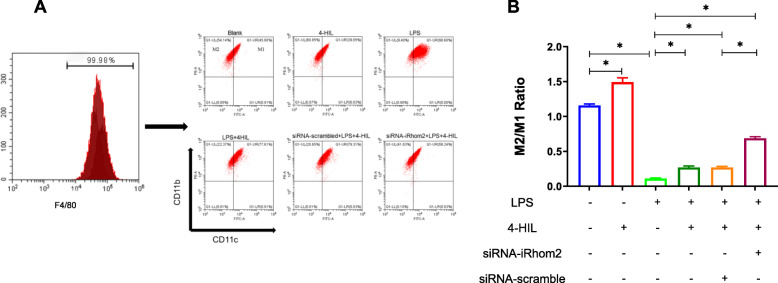
4-HIL promotes M2 macrophage polarization and inhibits M1 macrophage polarization in the co-culture system with LPS stimulation. **a** The number of M1 macrophages and M2 macrophages determined by flow cytometry. **b** The ratio of M2/M1. Quantitative data were presented as mean ± SD. ^*^P < 0.05

The ratio of M2/M1 was detected by flow cytometry (Fig. 6). Our findings showed that the ratio of M2/M1 was significantly decreased in the co-culture system (*P* < 0.05)*.* Meanwhile, the decrease of the M2/M1 ratio induced by LPS was noticeably reversed by 4-HIL (*P* < 0.05). Subsequently, these effects of LPS were remarkably abrogated by siRNA-iRhom2 transfection in RAW264.7 cells (*P* < 0.05). In addition, 4-HIL increased the ratio of M2/M1 without LPS stimulation (*P* < 0.05).

## Discussion

It has been identified that iRhom2 is highly involved in obesity-induced inflammatory status. Our previous studies demonstrated that 4-HIL, which is derived from *Trigonella foenum-graecum* L., significantly inhibited inflammation in adipocytes and hepatocytes by inhibiting TACE expressions. In light of the key role of iRhom2 in TACE-mediated proteolytic shedding of TNF-α, we hypothesized whether 4-HIL could modulate iRhom2 to reverse obesity-induced inflammation. In this study, the Transwell system was used to co-culture RAW264.7 macrophages and differentiated 3 T3-L1 adipocyte to mimic the inflammation microenvironment of obese adipose tissue. As expected, we found that 4-HIL markedly suppressed the mRNA and protein levels of iRhom2 and TACE in the co-culture system. Meanwhile, the pro-inflammatory cytokines (such as TNF-α, MCP-1 and IL-6) were remarkably decreased, and the anti-inflammatory cytokines (such as IL-10) were remarkably increased, indicating the anti-inflammatory properties of 4-HIL. Additionally, the inflammatory suppression of 4-HIL was confirmed by increasing the ratio of M2/M1, namely promoting the polarization of M2 macrophages and inhibiting the polarization of M1 macrophages. Finally, we showed that the inhibitory effect of 4-HIL on inflammation was more significant under the loss of iRhom2. This study suggested that 4-HIL is a promising anti-inflammatory agent in vitro model of obesity-induced inflammation.

iRhom2, also known as Rhbdf2, is a proteolytically inactive member of the seven transmembrane family of Rhomboid serine proteases. iRhom2 has a long N-terminal cytoplasmic tail that is predicted to be intrinsically disordered, decorated with signatures of a signaling hub, and multiple potentially phosphorylated residues [33–35]. iRhom2 plays a crucial role in the maturation and transport of TACE and the hydrolysis of the extracellular hydrolysis of TNF-α [16, 17]. iRhom2 gene-deficient mice exhibit reduced levels of TNF-α [18–22]. Our previous study indicated that 4-HIL inhibited inflammation in HepG2 cells and 3 T3-L1 adipocytes by decreasing TACE and TNF-α [26–28]. TNF-α is recognized as a major culprit in the chronic low-grade inflammatory state of obesity [36]. Hence, iRhom2 has become an important target for the treatment of obesity-induced inflammation. We questioned whether 4-HIL could inhibit inflammation by downregulating the expression of iRhom2. To solve this problem, we tested the mRNA and protein levels of iRhom2/TACE and tested the levels of TNF-α by ELISA in the co-culture system. 4-HIL reversed obesity-induced increased expression of iRhom2/TACE, and additionally reduced the levels of TNF-α in a dose-dependent manner. Furthermore, the mRNA and protein levels of iRhom2 decreased after the transfection of siRNA interference, and loss of iRhom2 further enhanced the effect of 4-HIL on iRhom2/TACE and TNF-α.

MCP-1 is secreted by a variety of cells, including epithelial cells, endothelial cells, smooth muscle cells, fibroblasts, and monocytes. It can recruit monocytes, memory T cells, and dendritic cells to damaged tissues or infected lesions [37]. MCP-1 had been shown to contribute to the infiltration of macrophages into adipose tissue, leading to obesity-induced inflammation [38]. Furthermore, the expression of MCP-1 in white adipose tissue and plasma was increased in obese mice [39]. Compared with the lean control group, the expression of MCP-1 was also significantly increased in obese patients [40–42]. Thus, we proposed whether 4-HIL might exert its functions on inflammations via decreasing the levels of MCP-1. To this end, we tested the levels of MCP-1 by ELISA in the co-culture system. Our findings indicated that 4-HIL dose-dependently decreased the levels of MCP-1, and the loss of iRhom2 further enhanced the effect of 4-HIL on MCP-1.

According to different activation states and functions, macrophages can be roughly divided into M1 type, which classically activates pro-inflammatory macrophages and M2 type, which is alternatively activated anti-inflammatory macrophages [43]. Macrophages have a series of continuous functional states, and M1 and M2 macrophages are two extremes of this continuous state. M1 macrophages release pro-inflammatory and chemokines, such as TNF-α, IL-6, and MCP-1, which can directly promote the migration of macrophages and further promote obesity-induced inflammation. In contrast, M2 macrophages secrete anti-inflammatory factors, such as IL-10, which suppress inflammation and have a positive effect on improving insulin resistance [30, 31]. Thus, we proposed whether 4-HIL might inhibit inflammation by affecting the migration and phenotype of macrophage. Then, the levels of IL-6 and IL-10 were tested by ELISA, and the ratio of M2/M1 was tested by the flow cytometer. Our findings showed that 4-HIL inhibited the migration of RAW264.7 macrophages into 3 T3-L1 adipocytes, lowered the levels of IL-6, and increased the levels of IL-10, as well as increased the ratio of M2/M1. Furthermore, the loss of iRhom2 further enhanced the effect of 4-HIL.

LPS is a component of the outer cell wall of Gram-negative bacteria, consisting of lipids and polysaccharides (glycolipids). LPS has a variety of biological activities, the most important of which is to trigger cell activity signaling through toll-like receptor 4 (TLR-4), which leads to the infiltration and activation of macrophages involved in the innate response [44]. LPS activates a cascade of signaling pathways in inflammation by recognizing TLR-4 in immune cells and other types of cells, such as adipocytes [45]. In obesity, LPS comes from excessive nutrients, such as saturated/free fatty acids, and enterotoxins to activate TLR4 and other Toll-like receptors, leading to severe inflammation and adipocytes dysfunction [46], suggesting that LPS can be an ideal stimulator for studying obesity-induced inflammatory response.

## Conclusions

Taken together, 4-HIL modulated the obesity-induced inflammation. It is worth noting that the underlying mechanism of its anti-inflammatory activity is that 4-HIL downregulated the pro-inflammatory cytokines (such as MCP-1 and IL-6) and upregulated the anti-inflammatory cytokines (such as IL-10). In particular, for the first time, we demonstrated that 4-HIL could suppress TNF-α production by reducing the expressions of iRhom2, which have been proved to be promising targets of obesity-related metabolic diseases. Our results suggested that 4-HIL could potently relieve LPS-induced inflammation through an iRhom2-dependent pathway. Besides, more efforts should be made to translate the current significance of 4-HIL into clinical applications.

## Supplementary Information

**Additional file 1: Figure S1.** Oil red O staining of preadipocytes cell slides (100X). (A-D) Cell morphology on day 3, 5, 7, and 9, respectively.

**Additional file 2: Figure S2**. Molecular structure of 4-HIL.

**Additional file 3.** Original western blots for Figs. 2 and 3.

## Data Availability

The datasets used or analyzed during the current study are available from the corresponding author on reasonable request. All data generated during this study are included in this manuscript.

## Abbreviations

4-HIL: 4-Hydroxyisoleucine
TNF-α: Tumor necrosis factor α
TACE: TNF-α converting enzyme
iRhom2: Inactive rhomboid protein 2
MCP-1: Monocyte chemotactic protein 1
LPS: Lipopolysaccharide
ELISA: Enzyme-linked immunosorbent assay
RT-qPCR: Real-time quantitative PCR
TLR-4: Toll-like receptors 4
JNK: c-Jun N-terminal kinase
MAPK: Mitogen-activated protein kinase
NF-κB: Nuclear factor kappa-B
