# Supplementary figures and images for: 4-Hydroxyisoleucine relieves inflammation through iRhom2-dependent pathway in co-cultured macrophages and adipocytes with LPS stimulation
Source: BMC Complement Med Ther. 2020 Dec 9;20:373. doi: 10.1186/s12906-020-03166-1 (PMC7724822; doi:10.1186/s12906-020-03166-1)

## Slide 1
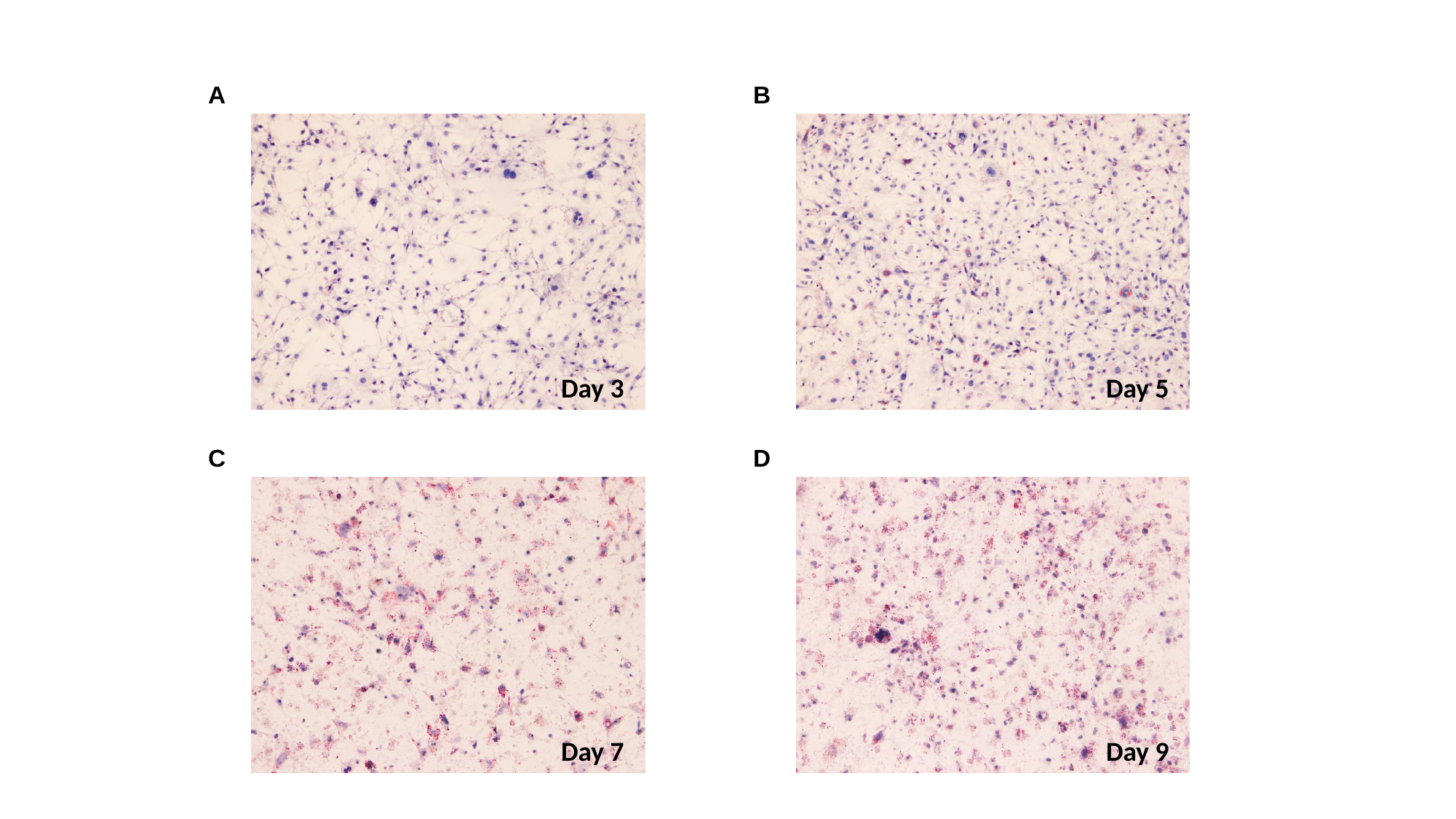

A
B
Day 3
Day 5
C
D
Day 7
Day 9

Supplement: Supplementary file 1 — Additional file 1: Figure S1. Oil red O staining of preadipocytes cell slides (100X). (A-D) Cell morphology on day 3, 5, 7, and 9, respectively. [file 12906_2020_3166_MOESM1_ESM.pptx]

## Slide 1
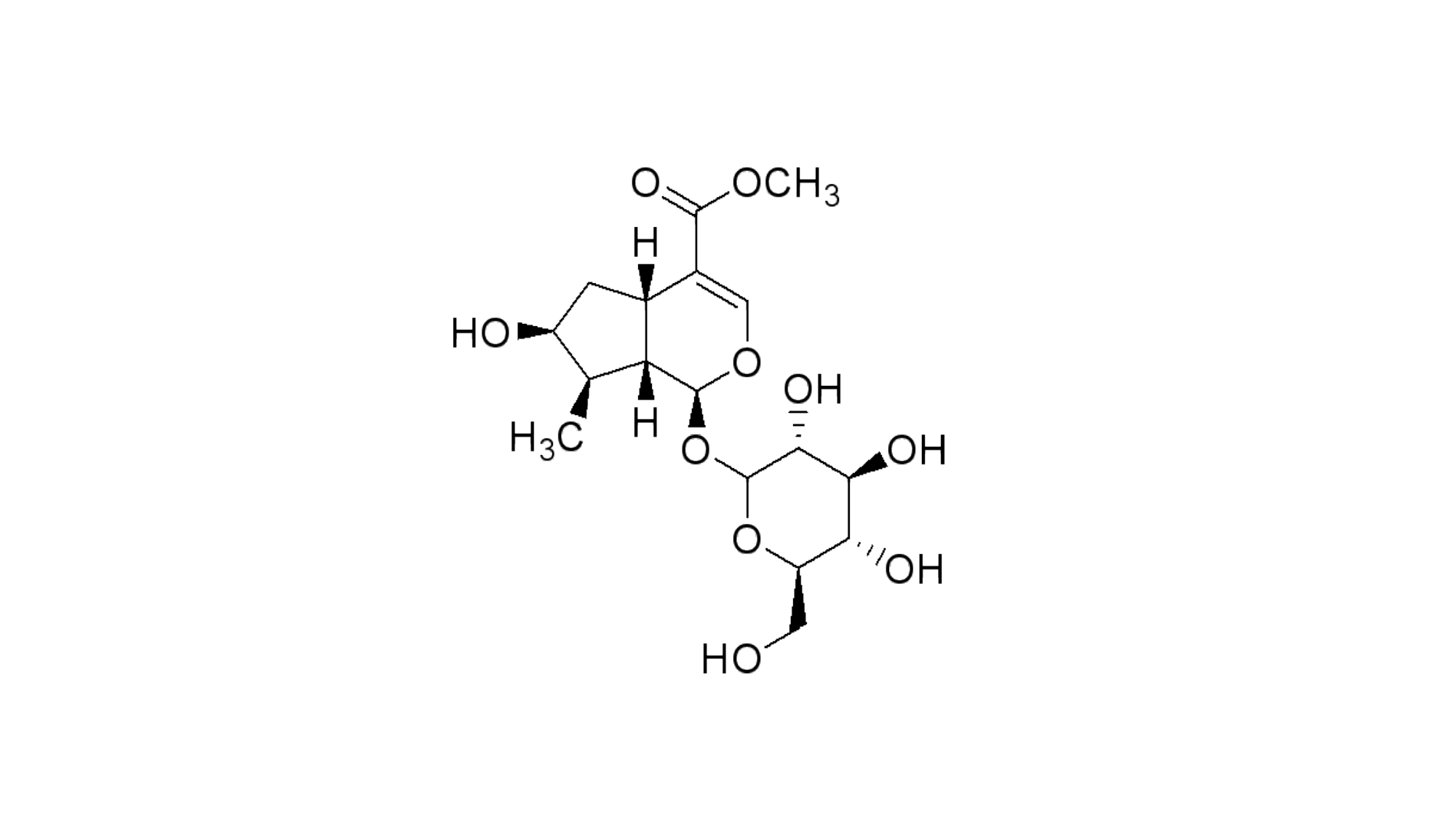

Supplement: Supplementary file 2 — Additional file 2: Figure S2. Molecular structure of 4-HIL. [file 12906_2020_3166_MOESM2_ESM.pptx]
