# Supplementary material for: 4-Hydroxyisoleucine relieves inflammation through iRhom2-dependent pathway in co-cultured macrophages and adipocytes with LPS stimulation
Source: BMC Complement Med Ther. 2020 Dec 9;20:373. doi: 10.1186/s12906-020-03166-1 (PMC7724822; doi:10.1186/s12906-020-03166-1)
Supplement: Supplementary file 3 — Additional file 3. Original western blots for Figs. 2 and 3. [file 12906_2020_3166_MOESM3_ESM.pptx]

## Slide 1
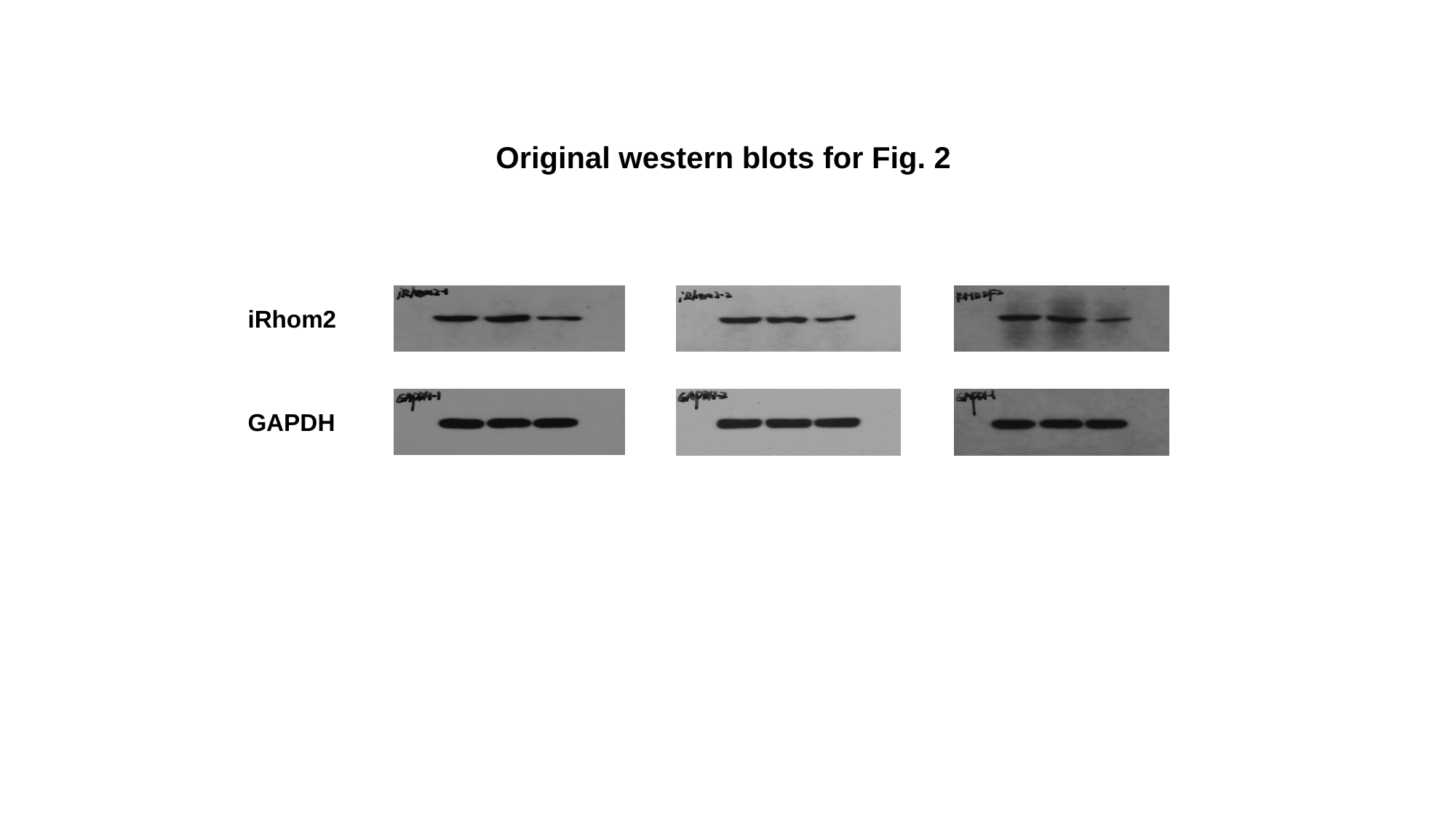

Original western blots for Fig. 2
iRhom2
GAPDH

## Slide 2
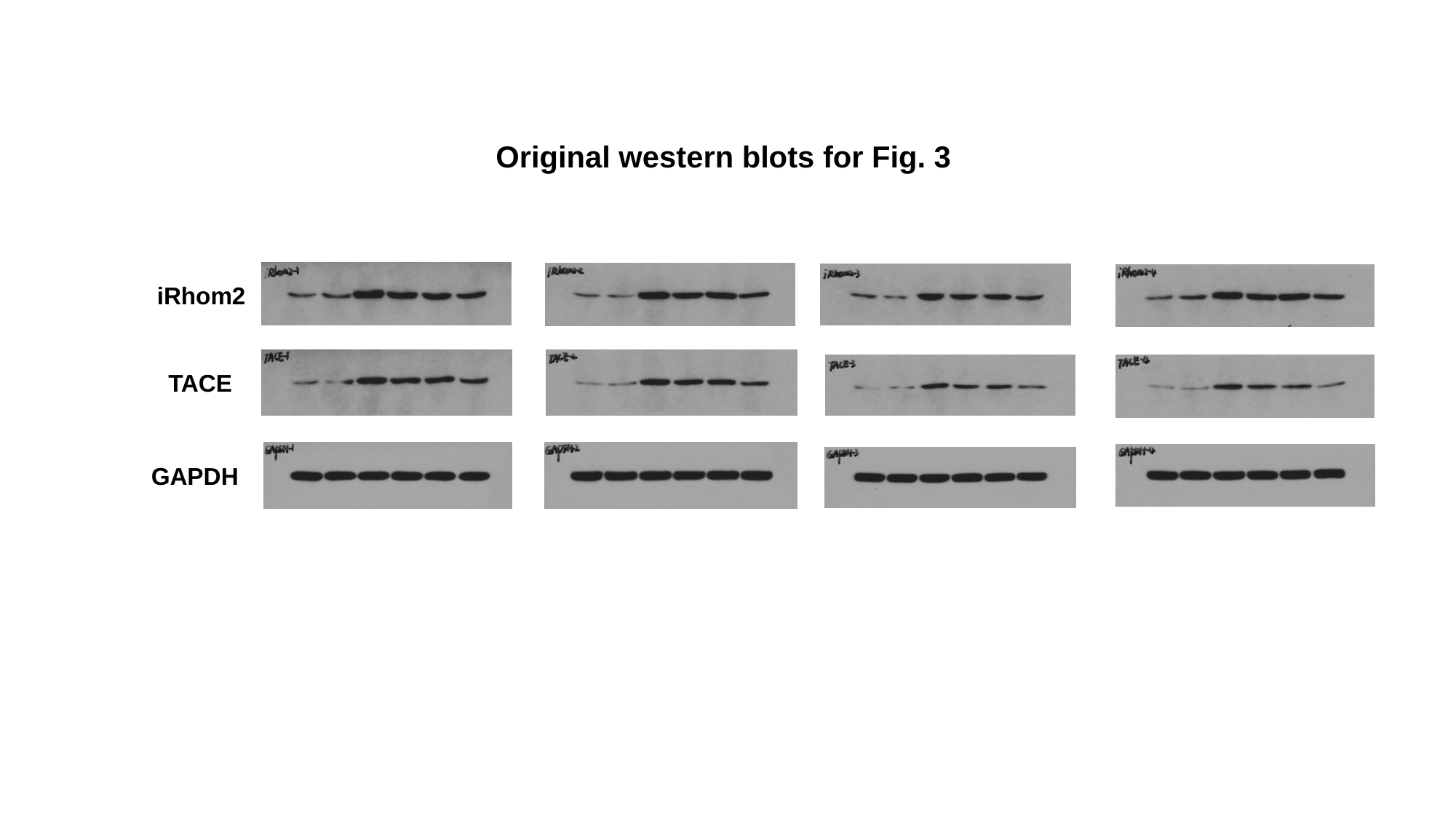

Original western blots for Fig. 3
iRhom2
TACE
GAPDH
